# Long non-coding RNAs in normal and malignant hematopoiesis

**DOI:** 10.18632/oncotarget.9308

**Published:** 2016-05-11

**Authors:** Lucia Nobili, Marta Lionetti, Antonino Neri

**Affiliations:** ^1^ Department of Oncology and Hemato-Oncology, Università degli Studi di Milano, Hematology, Fondazione IRCCS Cà Granda Ospedale Maggiore Policlinico, Milano, Italy

**Keywords:** long non-coding RNAs, hematopoiesis, hematological malignancies, transcriptional regulation, translation regulation

## Abstract

Long non-coding RNAs (lncRNAs) are defined as ncRNAs of more than 200 nt in length. They are involved in a large spectrum of biological processes, such as maintenance of genome integrity, genomic imprinting, cell differentiation, and development by means of mechanisms that remain to be fully elucidated. Besides their role in normal cellular physiology, accumulating evidence has linked lncRNA expression and functions to cancer development and progression. In this review, we summarize and discuss what is known about their expression and roles in hematopoiesis with a particular focus on their cell-type specificity, functional interactions, and involvement in the pathobiology of hematological malignancies.

## INTRODUCTION

In last years, after human genome sequencing, it became evident that although over 90% of the genome is actively transcribed [1, 2], the majority of transcripts is represented by non-coding RNA (ncRNA) and therefore not translated into canonical functional proteins. NcRNAs are represented by many classes, such as small interfering RNAs, microRNAs (miRNAs), PIWI-associated RNAs, small nucleolar RNA, and long ncRNAs (lncRNAs), including transcribed ultra-conserved regions (UCRs) and circular RNAs. NcRNAs are broadly divided into short (<200 nt) and long (>200 nt) transcripts. Dysregulation of short ncRNAs, particularly miRNAs, occurs in virtually all types of cancer, and this highlights the usefulness of miRNA profiling in diagnosis and prognosis, and in predicting response to therapy [3, 4]. Notably, they are currently considered both emerging therapeutic targets and innovative intervention tools in cancer, including hematological malignancies [5–7]. Among miRNAs with a relevant role in hematologic malignancies, we should mention miR-29 family members (miR-29a, miR-29b and miR-29c), reported to be widely dysregulated in hematologic cancers and demonstrated to target a number of epigenetic effectors, as reviewed by Amodio et al. [8].

**Figure 1:**
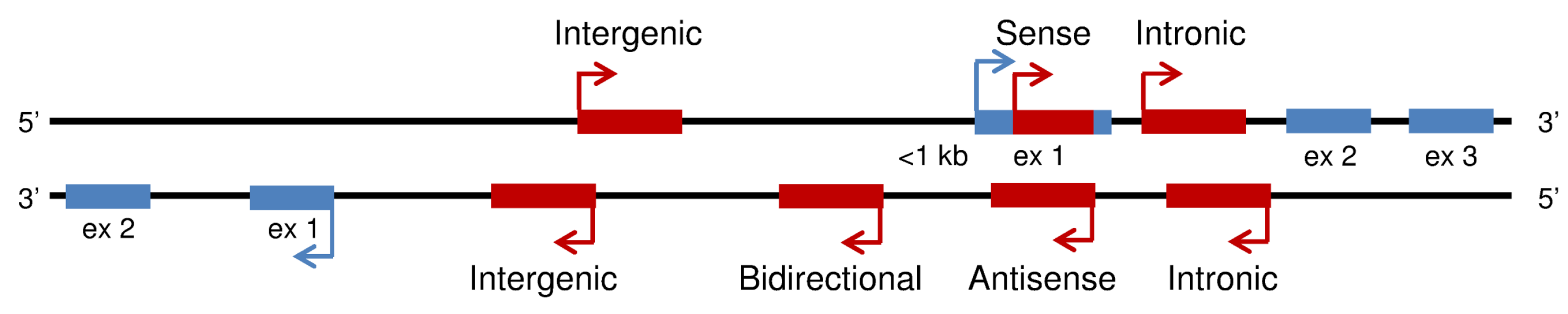
Categories of long non-coding RNA Overview of five broad categories of lncRNAs (sense, antisense, bidirectional, intronic, intergenic; depicted in red) based on their location relative to nearby protein-coding genes (depicted in blue).

**Figure 2:**
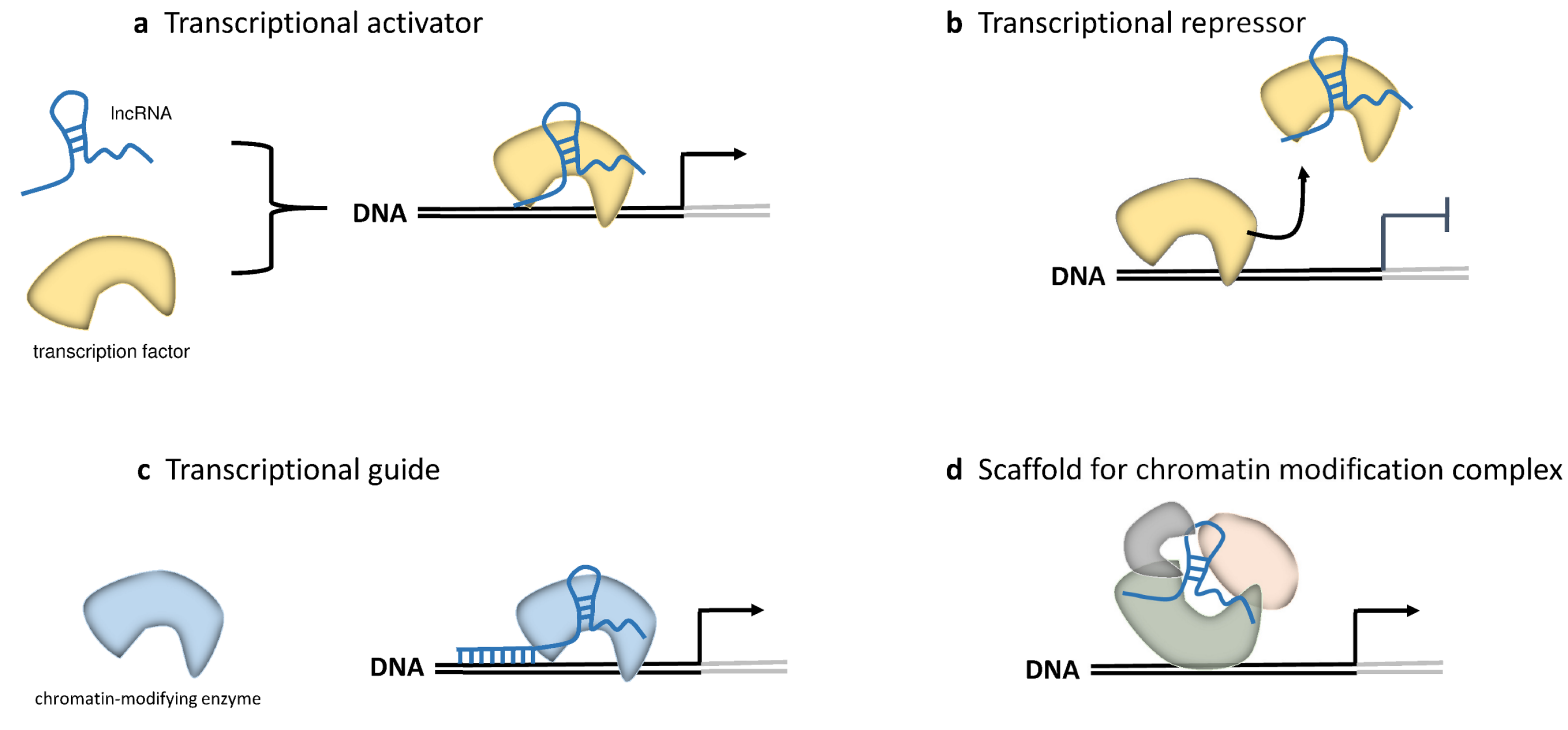
LncRNAs in epigenetic and transcriptional regulation Four mechanisms of epigenetic and transcriptional regulation by lncRNAs are shown [18]. **a.** Direct interaction of lncRNAs with transcription factors (TFs) induces the allosteric change of the TFs towards activation. **b.** LncRNAs act as decoy for TFs by keeping them away from their targets on chromatin. **c.** LncRNAs act as a transcriptional guide by recruiting chromatin-modifying enzymes to target genes, either in *cis* or in *trans* to distant target genes. **d.** LncRNAs act as a scaffold, bringing together multiple proteins to form ribonucleoprotein complexes.

**Figure 3:**
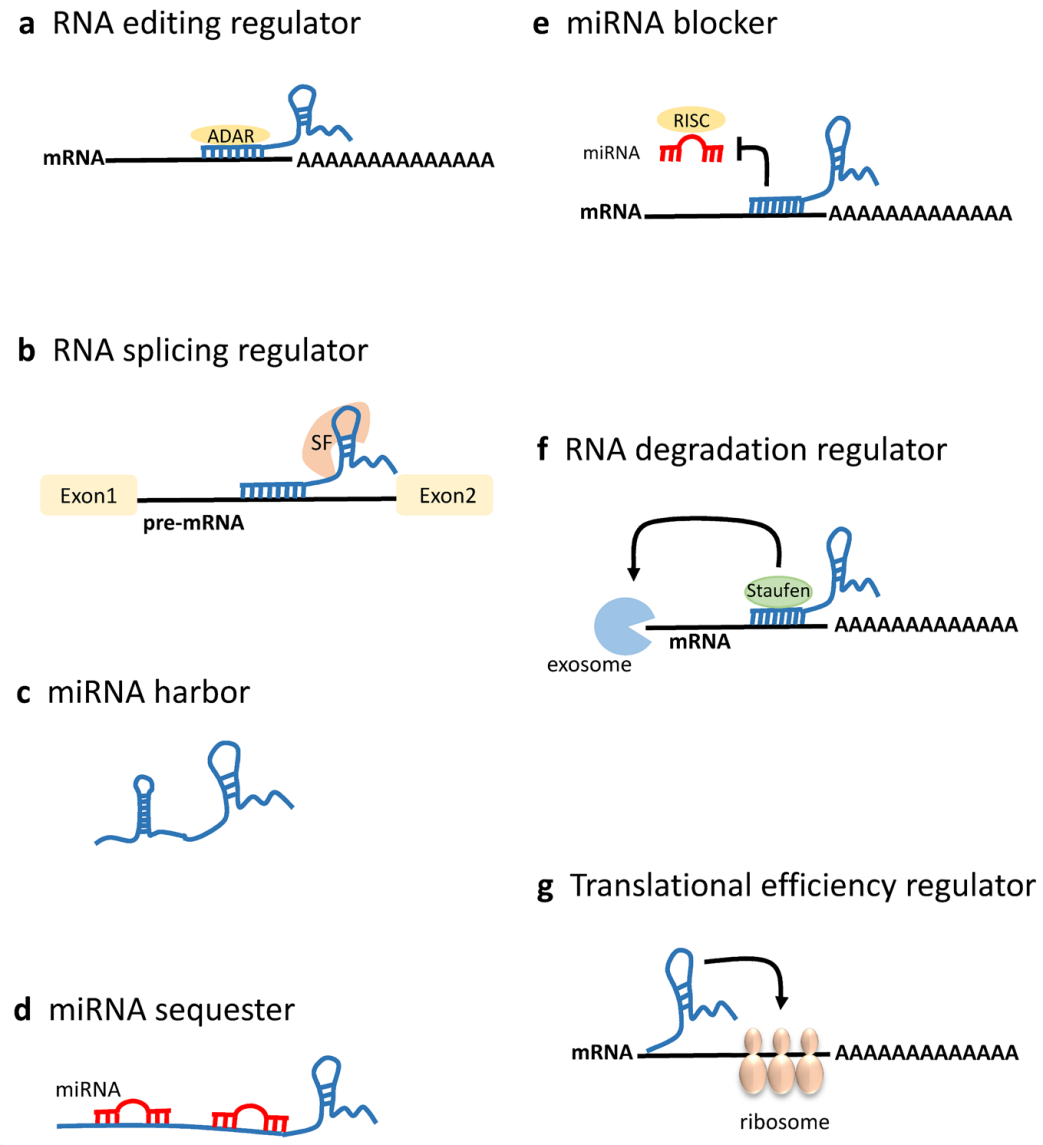
LncRNAs in mRNA processing and post-transcriptional regulation LncRNAs can act post-transcriptionally modulating mRNA processing at multiple levels [19]. **a.** Antisense lncRNAs associate with the sense mRNA, and the resultant RNA:RNA duplex might direct mRNA editing recruiting ADAR (adenosine deaminase acting on RNA) enzymes that catalyze adenosine to inosine conversion in double-stranded RNA. **b.** LncRNAs can prevent the alternative splicing of a pre-mRNA by binding the boundary site between its intron and exon. LncRNAs can also regulate RNA splicing by associating with splicing factors. **c.** LncRNAs may harbor the hairpin structure, which can give rise to the pre-miRNA. **d.** LncRNAs harboring the recognition site for functional miRNAs can function as miRNA decoys to sequester miRNAs from their mRNA targets. Furthermore, lncRNAs themselves can be the targets of miRNAs. **e.** LncRNAs can compete with miRNAs for binding on target mRNAs thus blocking miRNA-induced silencing through the RNA-induced silencing complex (RISC) and increasing mRNA translation. **f.** LncRNAs can regulate mRNA stability forming lncRNAs:mRNA double-stranded structures that can direct exosome mediated RNA degradation. For instance, Alu repeat-containing lncRNAs can associate with the Alu elements in the 3′ untranslated region (UTR) of an mRNA, and the resultant double-stranded structure can direct Staufen-mediated decay, thus destabilizing the target mRNA. **g.** LncRNAs association with the mRNA can positively or negatively modulate the translation efficiency, depending on the mRNA and lncRNA structures.

LncRNAs are a heterogeneous group representing more than half of the mammalian non-coding transcriptome. They have an overall relative lower level of sequence conservation among mammalian species than the protein coding genes [9]. However, this finding does not directly imply lack of function, since most lncRNAs fold into complex secondary and tertiary structures important for their biological activity [10, 11]. Indeed, an improved algorithm identified more than four million of RNA secondary structures conserved in mammalian genomes, 88% of which falling outside of known sequence-based conservation sites [12]. LncRNAs have been shown to be aberrantly expressed in cancer tissues and to be involved in oncogenic or tumor suppressive processes [13]. They are developmentally regulated and tissue-specific, and have been associated with a spectrum of biological processes, such as maintenance of genome integrity, X-chromosome inactivation, genomic imprinting, cell differentiation, and development. LncRNAs may also be defined based on their location relative to nearby protein-coding genes. According to this, a lncRNA can be placed into one or more of five broad categories: (1) sense, or (2) antisense, when overlapping with one or more exons of another transcript on the same, or opposite strand, respectively; (3) bidirectional, when the sequence is located on the opposite strand from a protein coding gene whose transcription is initiated less than 1000 base pairs away; (4) intronic, when it is derived from within an intron of a different coding transcript in either sense or antisense orientation; or (5) intergenic (known as lincRNA), when it lies within the genomic interval between two coding genes at least 1kb away from the nearest coding gene [14–16] (Figure 1). The number of known human lncRNA transcripts is still evolving. LNCipedia v3.1 contains some 111,685 human annotated lncRNAs, with many loci generating multiple transcripts [17]. LncRNAs exhibit a structure and biogenesis that do not differ greatly from mRNAs. There are some lncRNAs that are transcribed by RNA polymerase III while the majority of lncRNAs are transcribed by RNA polymerase II, spliced and polyadenylated [18]. Compared to mRNAs, most lncRNAs localize preferentially to the nucleus, are more cell-type specific and are expressed at lower levels [2]. The mechanisms underlying the function of most lncRNAs are not fully understood. They may regulate gene transcription and translation through different mechanisms, such as interaction with RNA-binding proteins to reduce the translation activity of mRNA, protein complex recruitment to epigenetically regulate gene expression, or competition with mRNAs for miRNA binding [18–23] (Figures 2 and 3).

Dysregulation of distinct lncRNAs has been reported to promote tumor formation, progression, and metastasis in many types of cancer [24, 25]. Moreover, accumulating evidence suggests that lncRNAs have multiple functions in normal and malignant hematopoiesis [26–32], which can help to better understand the biology of hematopoiesis and blood diseases. In this review, we summarize what is known about lncRNAs in normal hematopoiesis and in hematological tumors. The data available thus far indicate that several lncRNAs may be key molecules in hematopoiesis and in the pathogenesis of hematological malignancies, and suggest the potential clinical relevance of lncRNAs in the diagnosis, prognosis, and therapy of these diseases.

## LNCRNAS IN NORMAL HEMATOPOIESIS

**Figure 4:**
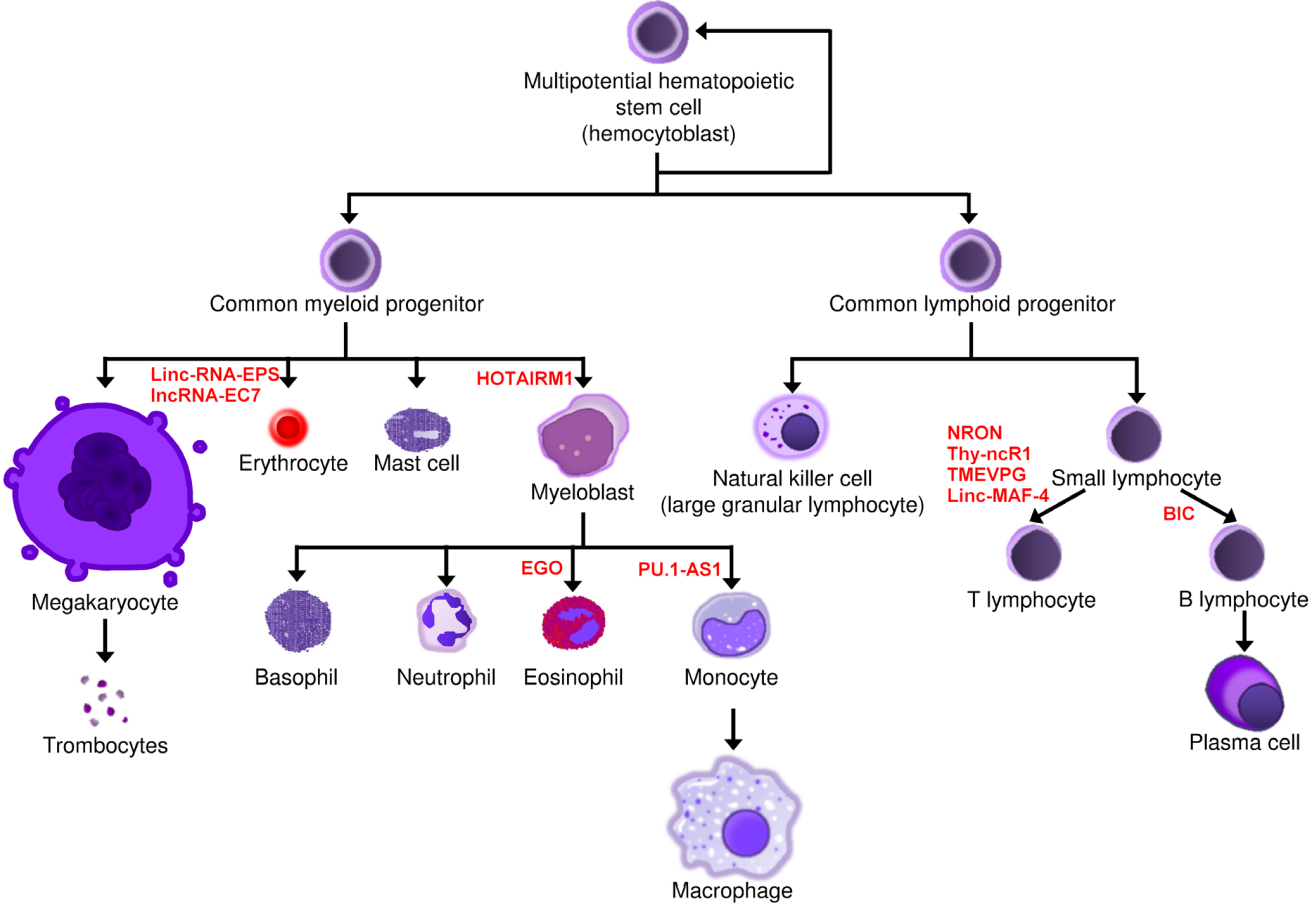
Involvement of lncRNAs in normal hematopoiesis LncRNAs that regulate blood cell development are shown next to the cellular stage at which they act.

Several lineage-specific lncRNAs have been already identified in the development of blood cells, although most of them have not yet been functionally characterized. Their physiological expression ensures the normal differentiation of hematopoietic stem cells and contributes to maintaining normal hematopoiesis (Figure 4, Table 1).

**Table 1:** LncRNAs with roles in normal hematopoiesis

lncRNA	Cell Type	Function	Reference(s)
EGO	Eosinophils	Regulator of major basic protein (*MBP*) and eosinophil derived neurotoxin (*EDN*) mRNA expression during eosinophil differentiation of CD34+ hematopoietic progenitor cells	[26]
PU.1-AS	Monocytes; macrophages	Negative regulator of the hematopoiesis regulator *PU.1* mRNA translation	[35, 36]
HOTAIRM1	Myeloid progenitors	Regulator of the neighboring 3′ *HOXA* genes and other granulocytic differentiation genes	[27, 37, 69]
lincRNA-EPS	Erythroid progenitors	Anti-apoptotic activity on erythroid precursor, at least in part functionally related to the inhibited expression of the pro-apoptotic gene *Pycard*	[29]
lncRNA-EC7	Erythroid progenitors	Activator of the neighboring gene encoding BAND 3, the major anion transporter of the red cell membrane, probably acting as an enhancer RNA during erythrocyte maturation	[33]
NRON	T cells	Regulator of dephosphorylation of the nuclear factor of activated T-cells 1 (NFAT1) transcription factor in the context of an RNA-protein scaffold complex	[39]
Thy-ncR1	Thymic T cells	Regulator of T-cell selection and maturation, probably by indirectly controlling the degradation of *MFAP4* (microfibril-associated glycoprotein 4) mRNA	[28]
TMEVPG1	T cells	Regulator of T-cell differentiation implicated in the transcription regulation of the *IFN-γ* gene	[41, 42]
linc-MAF-4	T cells	Regulator of CD4^+^ helper T-cell differentiation mediating the repression of *MAF* transcription in T_H_1 lymphocytes	[44]
BIC	B cells	Regulator of B-cell differentiation containing the mature miR-155 sequence	[45–47, 49]

### LncRNAs involved in erythroid differentiation

The first lncRNA to be related to red blood cells was the murine Erythroid ProSurvival lincRNA (lincRNA-EPS), identified by RNA-sequencing as highly specific among approximately 400 lncRNAs with modulated expression during red blood cells differentiation [29]. Functional studies indicated that lincRNA-EPS is highly induced in erythroid precursors when they start synthesizing hemoglobin and other lineage-specific proteins. Knocking-down lincRNA-EPS in mouse inhibited differentiation and promoted apoptosis of erythroid precursors, while its ectopic expression could prevent this process. It has been suggested that the anti-apoptotic activity of lincRNA-EPS on erythroid progenitors might be at least in part functionally related to the inhibited expression of the pro-apoptotic gene *Pycard* through a still not defined mechanism [29].

In a recent high-throughput RNA-sequencing study of differentiating mouse fetal liver erythroid cells [33], Alvarez-Dominguez et al. identified more than a hundred not previously annotated lncRNA transcripts with erythroid-restricted expression. Many of them were targeted by key erythroid transcription factors, such as GATA1, TAL1, or KLF1, strongly supporting their roles during erythropoiesis. The deep investigation of twelve of these lncRNAs revealed that they were localized in the nucleus and exhibited complex patterns of expression during developmental stages. Their silencing severely impaired erythrocyte maturation by affecting cell-size reduction and subsequent enucleation. In particular, the lncRNA-EC7, transcribed from an erythroid-specific enhancer, was required for the activation of the neighboring gene encoding BAND 3, the major anion transporter of the red cell membrane, probably acting as an enhancer RNA.

In a further study, Paralkar et al. used deep sequencing of polyadenylated RNAs to examine lncRNA expression in purified murine erythroblasts, megakaryocyte-erythroid precursors (MEPs), megakaryocytes, and in human erythroblasts [34]. Interestingly, they defined several lncRNAs unique to each cell type, demonstrating that most of them are transcribed from H3K4me3-rich gene promoters and are regulated by the same key transcription factors networks, including GATA1 and TAL1. Focusing on the erythroid lineage, they demonstrated that lncRNA expression was largely conserved among eight different mouse strains. In contrast, even though orthologous regions were identified for about 90% of the lncRNA genes, only 15% of erythroid lncRNA genes expressed in mouse erythroblasts were also expressed in human erythroblasts at similar developmental and maturational stages, and vice versa. These findings reflect a marked species-specificity of lncRNAs. Furthermore, the Authors identified seven transcripts whose knockdown inhibited terminal maturation of primary mouse erythroid precursors. Of note, six of these seven lncRNAs had no detectable expression in human erythroblasts, even though orthologous genomic loci were identified. These findings suggest that the lack of expression of conserved lncRNA genes between mammalian species does not necessarily predict their biological inactivity.

### LncRNAs involved in myeloid differentiation

One of the first lncRNAs related to normal hematopoiesis to be identified was Eosinophil Granule Ontogeny (EGO), highly expressed in human bone marrow and in mature eosinophils [26]. This lncRNA was identified in eosinophil differentiation of CD34+ hematopoietic progenitor cells, where it regulates the mRNA expression of major basic protein (MBP) and eosinophil derived neurotoxin (EDN). EGO is transcribed antisense within an intron of the inositol triphosphate receptor type 1 (*ITPR1*) gene, and is conserved at the nucleotide level, with up to 90% identity among mouse, human, and chicken (http://genome.lbl.gov/vista) [26]. However, its mechanism of action has not been understood yet.

The lncRNA PU.1-AS is transcribed antisense to the transcription factor PU.1, with both sense and antisense transcripts originating from the same promoter. PU.1 is an essential regulator for normal hematopoiesis; its precise expression levels are crucial for specifying cell fate, and, if perturbed, can lead to leukemias and lymphomas [35]. Moreover, PU.1 plays a critical role in monocytes lineage commitment and monocyte/macrophage maturation [36]. PU.1-AS was demonstrated to negatively regulate *PU.1* mRNA translation in murine and human cell lines by a mechanism similar to miRNAs, i.e. binding to *PU.1* mRNA to form mRNA/AS lncRNA duplex, thus antagonizing the expression of PU.1 protein [35].

One of the best-studied lncRNAs involved in hematopoiesis is HOTAIRM1 (HOX antisense intergenic RNA myeloid 1), a lincRNA located between the human *HOXA1* and *HOXA2* genes. HOTAIRM1 is specifically expressed in the myeloid lineage, most highly in the terminal stage of granulocytic differentiation [27]. HOTAIRM1 was identified by microarray analysis as one of the lncRNAs induced during all-trans retinoid acid (ATRA)-driven granulocytic differentiation of the NB4 human acute promyelocytic leukemia cell line and normal myeloid lineage hematopoietic cells. Knockdown of HOTAIRM1 attenuated ATRA-induced expression of neighboring 3′ *HOXA* genes including *HOXA1* and *HOXA4*, and selectively impaired the induction of transcripts for the myeloid differentiation markers CD11b (integrin alpha M chain), CD18 (integrin beta 2 chain) and CD11c (integrin alpha X chain), while retaining the expression of CD49d (integrin alpha 4 chain) [27, 37]. A resistance to ATRA-induced cell cycle arrest at the G1/S phase transition in knockdown cells was also observed [37], suggesting that the HOTAIRM1-modulated shift in CD49d and CD11c expression might function as a switch regulating the transit from the proliferative phase to granulocytic maturation.

### LncRNAs involved in lymphoid differentiation

The first genome-wide characterization of lncRNAs expressed in mammalian CD8^+^ T-cells [38] identified more than 1000 lncRNAs in human and mouse CD8^+^ T-cells, many of them displaying stage- or tissue-specificity. The expression of about 10% of these lncRNAs changed significantly during either CD8^+^ effector T-cells activation and/or naïve to memory cell differentiation. Furthermore, several of the lncRNAs neighbored protein-coding genes with well-characterized immunologically important roles in CD8^+^ T-cells, and/or overlapped shorter functional RNAs, suggesting that lncRNAs may be processed and exert their effects *via* smaller functional species.

Nuclear factor of activated T-cells (NFAT) proteins are Ca^2+^-regulated transcription factors controlling gene expression in many cell types. A study by Sharma et al. [39] in mouse CD8^+^ T-cells or HA-NFAT1 Jurkat T-cells, stably expressing HA (hemagglutinin)-tagged NFAT1-GFP, provided evidence that a cytoplasmic complex involving the lincRNA NRON [noncoding (RNA) repressor of NFAT] and the IQ motif-containing GTPase-activating protein 1 (IQGAP1) forms a scaffold for the inactive phosphorylated NFAT1 and its inhibitory kinases. Knockdown of linc-NRON in stimulated cells enhanced NFAT1 dephosphorylation and nuclear translocation, and increased production of NFAT-dependent cytokines. These data support the notion that lincRNAs may modulate gene expression by functioning as scaffolds for transcriptional regulators in the context of large RNA-protein complexes.

Another lncRNA related to T-cell differentiation, Thy-ncR1, is expressed specifically in the thymus, the site of T-lymphocyte maturation. Aoki et al. [28] identified Thy-ncR1 to be expressed only in a few human T-cell leukemia cell lines (DND41, HPB-ALL, Jurkat, and MOLT-3), all of which originated from immature stage III T-cells. The expression of the *CD1* gene cluster, located 118 kb from *Thy-ncR1* gene, was highly correlated with Thy-ncR1 expression, implying a potential synergistic effect of CD antigens and lncRNAs during T-cell selection and maturation, although it is unclear if the expression of Thy-ncR1 is mechanistically linked to the *CD1* gene cluster expression. In addition, the Authors suggested that Thy-ncR1 might participate in T-cell selection and maturation by an indirect control of the *MFAP4* (microfibril-associated glycoprotein 4) mRNA degradation [28]. *MFAP4* encodes a protein containing a fibronectin-like domain involved in cell adhesion. Although the role of MFAP4 in immature T-cells has not been defined yet, it was shown to support the *ex vivo* expansion of mouse hematopoietic stem cells [40].

*TMEVPG1* and its murine ortholog are lincRNA genes located adjacent to the interferon (IFN)-γ-encoding gene in both mouse and human genome (*IFNG*). TMEVPG1 RNA is encoded antisense to *IFN*-γ. Initially identified in the context of Theiler's virus infection in mice, the human ortholog TMEVPG1 is expressed in human NK cells and CD4^+^ and CD8^+^ T lymphocytes only when these subsets are not stimulated [41]. A study by Collier et al. [42] described TMEVPG1 and its murine ortholog as Th1-specific lincRNAs that require STAT4 and TBET transcription factors to drive the Th1 differentiation program, contributing to the transcription of the *IFN-*γ gene. Furthermore, in addition to increasing Theiler's virus persistence in activated CD8^+^ T lymphocytes [43], murine Tmevpg1 was found to bind WDR5, a component of the H3K4 methyltransferase complex, and to alter H3 methylation at the *Ifn-γ* locus. These findings suggest that lncRNAs may have a crucial role in T-cell differentiation and in susceptibility to infectious diseases acting as transcription regulators of pivotal cytokines.

A recent study by Ranzani et al. investigated lincRNAs in 13 highly purified human T and B lymphocytes subsets by RNA-sequencing and *de novo* transcriptome reconstruction [44]. They identified a lincRNA (linc-MAF-4) that seemed to have a key role in the differentiation of CD4^+^ helper T-cells. Indeed, the expression of this chromatin-associated lincRNA specific to the T_H_1 subset of helper T-cells was inversely correlated with expression of MAF, a T_H_2-associated transcription factor. Experimental downregulation of linc-MAF-4 skewed differentiating helper T-cells toward a T_H_2 transcription profile. The Authors suggested that linc-MAF-4 might regulate *MAF* transcription through the recruitment of the chromatin modifiers LSD1 and EZH2, exploiting a chromosome loop that brought its genomic region close to the *MAF* promoter. These results further demonstrate that lincRNAs can have a key role in T lymphocyte differentiation.

Among the few lncRNAs reported as acting in B-cell differentiation, it is worth to mention the B-cell integration cluster (BIC), even if a clear evidence of BIC acting as a “long” RNA has not been found yet [45, 46]. *BIC* consists of three exons spanning a 13 kb region at chromosome 21q21; it was found to be highly expressed in antigen receptor stimulated B- and T-cells as well as in macrophages and dendritic cells upon TLR (Toll-like receptor) stimulation. Interestingly, the studies on this non-coding RNA have been mainly focused on its processed products, miR-155-5p and miR-155-3p, which play a key role in several biological processes, including hematopoiesis, inflammation and immune responses [47]. According to large-scale cloning studies [48], the *BIC* gene has now been designated as MIR155 host gene or *MIR155HG* (http://www.genenames.org/) whereas the BIC transcript is identified as pri-miR-155. Interestingly, high levels of BIC and miR-155 have been found in Hodgkin's lymphoma, primary mediastinal B-cell lymphoma, diffuse large B-cell lymphoma, chronic lymphocytic leukemia (CLL), AML and some solid tumors, but they are not expressed in healthy samples [47], indicating that this locus may be linked to cancer. Concerning miR-155, we should note that it has been reported by Calin et al. [49] to target other types of non-coding RNA, such as genomic transcribed UCRs. Interestingly, Petri et al. [50] have recently analyzed lncRNA expression during human B-cell development by array-based expression profiling of eleven distinct B-cell subsets isolated by flow cytometry cell-sorting from human tonsils and bone marrow. By means of a weighted gene co-expression network analysis, they identified several lncRNAs within well-defined gene networks involved in specific stages of B-cell development, such as early B-cell development, B-cell proliferation, affinity maturation of antibody, and terminal differentiation. These findings may be an important resource for future studies exploring the functions of lncRNAs in normal B-cell lymphopoiesis. Moreover, they might provide the basis for understanding the roles of lncRNAs in the pathogenesis and progression of B-cell malignancies that originate from distinct B-cell subpopulations.

## LNCRNAS DYSREGULATED IN HEMATOLOGICAL MALIGNANCIES

The central role of lncRNAs in regulating blood cell fates, including differentiation, proliferation and survival, suggests that they might be involved in the pathogenesis of hematopoietic malignancies. Whatever the mechanism(s) through which lncRNAs execute their functions, they may contribute to disease by misregulating target genes or signaling pathways crucial for disease onset and progression. Thus far, only few lncRNAs have been conclusively linked to the initiation and progression of hematological malignancies, though the list is expected to grow exponentially in the near future (Table 2).

**Table 2:** Overview of the most frequently deregulated lncRNAs in hematological malignancies

lncRNA	Hematologic disease	Function	Molecular mechanism	Reference(s)
XIST	MPN, MDS	Tumor suppressor	Not described	[32, 51]
H19	CML, PV, ET, PMF, CMML, AML, adult T-cell leukemia/lymphoma	Oncogene/tumor suppressor	Activated by c-Myc. Precursor of miR-675 targeting *RB*	[52–58]
BGL3	CML	Tumor suppressor	Competitive endogenous RNA cross-regulating the expression of the tumor suppressor *PTEN*	[59]
IRAIN	AML	Tumor suppressor	Interaction with the promoter and enhancer regions of the *IGF1R* gene	[60, 61]
MEG3	AML; MDS; MM	Tumor suppressor	Regulation of the Rb-p16^INK4a^ pathway. P53 activation	[62–67]
LUNAR1	T-ALL	Oncogene	NOTCH1-regulated. Activation of *IGF1R* expression in *cis* by recruitment of the Mediator complex and RNA polymerase II to the *IGF1R* enhancer	[72]
DLEU2	CLL, MCL, MM	Tumor suppressor	NF-kB activation. Host of miR-15a/16-1 cluster targeting *BCL2*	[76, 78–85, 115]
ANRIL	ALL, AML	Oncogene	PRC1 and PRC2 recruitment to epigenetically silence *INK4b-ARF-INK4a* tumor suppressor locus	[31, 86–89]
GAS5	B-cell lymphoma, T-cell leukemia	Tumor suppressor	Glucocorticoid receptors antagonist. Regulated by mTOR pathway	[85, 90–101]
TUG1	MM, CLL	Oncogene	PRC2 binding to repress cell-cycle regulation genes. Induced by p53	[101, 102]
MALAT1	MM	Oncogene	Sp1 recruitment to the promoter of *LTBP3* gene regulating the bioavailability of TGF-β	[25, 85, 108–114, 116, 117]

*Abbreviations*: AML, acute myeloid leukemia; ALL, acute lymphoblastic leukemia; CLL, chronic lymphocytic leukemia; CML, chronic myeloid leukemia; CMML, chronic myelomonocytic leukemia; ET, essential thrombocythemia; MCL, mantle cell lymphoma; MDS, myelodysplastic syndrome; MM multiple myeloma; MPN, myeloproliferative neoplasm; PMF, primary myelofibrosis; PV, polycythemia vera

### LncRNAs in myeloproliferative diseases

X-inactive-specific-transcript (XIST), one of the several lncRNAs located on the X chromosome and participating in the X chromosome inactivation during embryogenesis, has long been associated with human cancer [51], but only recently it has been causally linked to the development of hematologic tumors in mouse models [32]. In fact, by deleting *Xist in vivo* in murine hematopoietic stem cells after the occurrence of X inactivation, Yildrim et al. were able to induce highly aggressive lethal myeloproliferative neoplasm and myelodysplastic syndrome (MPN/MDS), with 100% penetrance, in both the female homozygous and heterozygous conditions, whereas male mutants remained healthy and viable throughout the 2-year observation period [32]. These results suggested that Xist, in addition to be directly involved in the formation of repressive chromatin for dosage compensation, has also an important role in tumor suppression *in vivo*. Indeed, Xist loss resulted in X reactivation and the induction of genome-wide changes associated with cancer, including dysregulation of oncogenes or tumor suppressor genes involved in MPN and MDS [32].

H19 was the first imprinted lncRNA gene identified. Genomic imprinting is a form of epigenetic gene regulation that results in expression of a single allele in a parent-of-origin-dependent manner. H19, abundantly expressed during embryonic development and downregulated after birth, is transcribed from the *H19*/insulin-like growth factor 2 (*IGF2*) cluster: H19 is expressed from the maternal allele and *IGF2* from the paternal allele [52]. Dysregulation of lncRNA H19 has been observed in various tumors, and it was described acting either as an oncogene or a tumor suppressor [52]. Reduced expression of H19 was observed in clinically untreated chronic myeloproliferative disorders, including chronic myeloid leukemia (CML), polycythemia vera (PV), essential thrombocythemia (ET), primary myelofibrosis (PMF) and chronic myelomonocytic leukemia (CMML), as well as in AML [53, 54]. Interestingly, a critical requirement for H19 was recently demonstrated in Bcr-Abl-mediated tumorigenesis [55]. This lncRNA was found upregulated in human K562 Bcr-Abl-positive leukemia cell line and primary CMLs in a Bcr-Abl kinase-dependent manner. Downregulation of H19 expression in K562 cells affected cell survival and attenuated tumor formation in xenograft mouse model suggesting a functional involvement of H19 in Bcr-Abl-mediated cellular transformation. Noteworthy, loss of imprinting of H19, resulting in high H19 expression, was described in adult T-cell leukemia/lymphoma patients and cell lines [56], suggesting that H19 might play different roles in distinct hematological tumors. The fact that H19 is also the precursor of miR-675 [57], known to downregulate the retinoblastoma (RB) gene in human colorectal cancer [58], adds a further level of complexity to the functional roles that H19 may have in different signaling pathways and cell contexts.

Another lncRNA acting as a key regulator of Bcr-Abl-mediated cellular transformation is the Beta Globin Locus 3 (BGL3) [59]. This lncRNA was upregulated by disruption of Bcr-Abl expression or inactivation of Abl kinase after exposure to imatinib in K562 cell line and primary CML samples. It was also observed that the ectopic expression of BGL3 sensitized K562 leukemic cells to imatinib-induced apoptosis and inhibited Bcr-Abl-induced tumorigenesis in xenograft mouse model. Furthermore, transgenic mice expressing BGL3 exhibited impaired Bcr-Abl-mediated primary bone marrow transformation. These observations suggest that BGL3 might act as a tumor suppressor during Bcr-Abl-induced tumorigenesis. The Authors also demonstrated that BGL3 was a target of a set of miRNAs known to repress the tumor suppressor phosphatase and tensin homolog (*PTEN*) gene, then suggesting that BGL3 might function as a competitive endogenous RNA to cross-regulate *PTEN* expression [59].

IRAIN is a recently discovered imprinted lncRNA, expressed exclusively from the paternal allele within the insulin-like growth factor type I receptor (*IGF1R*) locus [60]. IRAIN is transcribed antisense to *IGF1R* and is involved in the formation and/or maintenance of a long-range intrachromosomal loop between the *IGF1R* promoter and a distant intragenic enhancer. IGF1R is known to play a critical role in AML, promoting cell growth in samples with an activated phosphoinositide 3 kinase (PI3K)/Akt signaling pathway [61]. Interestingly, IRAIN was found downregulated in leukemia cell lines and in patients with high-risk AML [60]. This observation suggests that IRAIN downregulation might relax the ‘transcription competition’ control, which may contribute to activate the *IGF1R* gene, leading to growth advantage and tumor progression.

The maternally expressed gene 3 (MEG3) is a lncRNA with a tumor suppression function mediated by both p53-dependent and p53-independent mechanisms [62]. The expression of the *MEG3* gene is under epigenetic control, and aberrant CpG methylation has been observed in several types of cancer. Indeed, MEG3 is commonly downregulated in many types of tumors including hematological malignancies such as AML, MDS or multiple myeloma (MM) and the hypermethylation of its promoter is a marker of poor prognosis [63–65]. Although no clear role in the etiology of AML has emerged for MEG3, it is likely that it may contribute to the disease *via* its known effects on cell proliferation through regulation of the RB and p16^INK4a^ pathway [66, 67].

Recently, Garzon et al. [68] investigated the associations of lncRNA expression with clinical features, recurrent mutations, and outcome in cytogenetically normal AML (CN-AML) patients. They identified distinctive lncRNA profiles associated with the most common recurrent mutations in CN-AML, such as *FLT3*-ITD and those affecting *NPM1*, *CEBP, IDH2*, and *RUNX1* genes. Interestingly, a lncRNA score was derived, which strongly correlated with treatment response and survival [68]. Patients with *NPM1* mutations showed one of the strongest lncRNA signatures with many up-regulated lncRNAs transcribed antisense to *HOX* genes, such as HOXB-AS3 and MEIS1-AS2. As discussed above, HOTAIRM1, a lncRNA transcribed antisense to the *HOXA* genes, has been suggested to play a role in myelopoiesis [27, 37]. Notably, *HOTAIRM1* was also found upregulated in a large series of AML patients with intermediate-risk cytogenetics, in particular in patients with *NPM1* mutation, showing an independent negative prognostic value [69]. These findings suggest a role of *HOX* genes in the context of AML with *NPM1* mutations.

### LncRNAs in lymphoproliferative diseases

NOTCH1 receptor signaling is known to play a central role in T-cell lineage commitment and in supporting the growth and proliferation of immature T-cell progenitors during lymphoid development in the thymus [70]. Human T-cell acute lymphoblastic leukemia (T-ALL) is generally associated with *NOTCH1* gene mutations that trigger an aberrant and constitutively active NOTCH1 signaling [71]. Recently, Trimarchi et al. [72] have shown that a specific NOTCH1-regulated lncRNA, LUNAR1 (LeUkemia-induced Non-coding Activator RNA-1), is upregulated in *NOTCH1* mutated T-ALL and is essential for efficient T-ALL growth *in vitro* and *in vivo* due to its ability to enhance the expression of its neighboring coding gene on chromosome 15, *IGF1R*, thus sustaining the IGF1 signaling. IGFR1 receptor has been previously suggested to mediate important growth/survival signals in T-ALL [73]. Through a chromatin loop, that places the *IGF1R* enhancer and the LUNAR1 promoter in close proximity, the intronic *IGF1R* enhancer activates the transcription of LUNAR1, which in turn co-occupies this enhancer and sustains the *IGF1R* expression and signaling [72].

Deleted in leukemia 1 (DLEU1) and 2 (DLEU2) are two lncRNAs whose genes map in critical region at chromosome 13q14.3 found to be deleted in more than 50% of CLL patients [74, 75]. *DLEU2* gene hosts miRNAs 15a and 16-1, a cluster having a crucial role in the pathogenesis of CLL, in part by regulating the expression of the oncogene B cell lymphoma 2 (*BCL2*) [76, 77]. Mir-15a and mir-16-1 precursors are localized in intron 4 of *DLEU2* gene, on the same chromosome strand. In a recent study, mice deleted for the entire minimal deleted region (MDR) within 13q14, comprising the *DLEU2* lncRNA gene, developed clonal B-cell proliferations recapitulating the spectrum of CLL-associated phenotypes observed in humans, and displayed a significantly more aggressive phenotype than miR-15a/16-1-deleted mice [78]. Another study showed that DLEU2 had a potent inhibitory effect on cellular proliferation and colony-forming ability of tumor cell lines in a miR-15a/16-1-dependent manner [79]. Furthermore, DLEU2 expression caused a clear downregulation of the Cyclin D1 and Cyclin E1 protein levels in a way dependent on the expression of miR-15a/16-1. Taken together, these findings have suggested that loss of DLEU2 might contribute to CLL through the absence or functional loss of miR15a/16-1, although additional, yet unexplained, roles for DLEU2 in CLL development might be hypothesized. Furthermore, *DLEU1* and *DLEU2* were found to be significantly demethylated at the 5′ ends in almost all of CLL patients characterized by Garding et al. [80]. Demethylation correlated with transcriptional deregulation of a cluster of neighboring protein-coding tumor suppressor genes, which may act as positive regulators of NF-kB activity. As no significant enrichment of DLEU1 or DLEU2 transcripts was found at chromatin level, the Authors suggested that the two lncRNAs regulate the neighboring cluster genes by divergent transcription. Interestingly, deletion of the chromosome 13q14 region is a common genetic aberration in other types of lymphoid malignancies, such as mantle cell lymphoma (MCL) [81, 82] and, in particular, MM [83, 84]. This may suggest a role for DLEU2 also in the development of these hematological diseases. A recent study by Ronchetti et al. [85] investigated lncRNA expression profiles in a large cohort of samples representing all the major different forms of plasma cell (PC) dyscrasias, including monoclonal gammopathy of undetermined significance (MGUS), smoldering MM (SMM), truly overt and symptomatic MM, and extra-medullary myeloma/plasma cell leukemia (PCL) patients. The Authors found a significant downregulation of DLEU2 in patients carrying del13; in addition, DLEU2 expression significantly correlated with that of miR-15a and miR-16-1.

ANRIL (Antisense Non-coding RNA in the *INK4* Locus) is a cell cycle-related lncRNA transcribed antisense to the *INK4b-ARF-INK4a* locus, which encodes three crucial cyclin-dependent kinase inhibitors, p15^INK4b^, p14^ARF^ and p16^INK4a^ [86]. These inhibitors undergo epigenetic silencing in hematopoietic stem cells and play central roles in cell cycle inhibition, senescence, differentiation, and stress-induced apoptosis [87]. ANRIL is a key regulatory molecule mediating human disease at different levels and cellular settings. A statistically significant association between an ANRIL polymorphism and Philadelphia positive ALL (Ph+ ALL) was observed [88]. Furthermore, an inverse correlation between ANRIL and p15 expression was discovered in patients with ALL and AML [31], suggesting an ANRIL-dependent regulation mechanism for p15 in leukemia. Indeed, a *cis*-regulation of the *INK4* locus by ANRIL leading to the subsequent silencing of this gene locus by H3K27-trimethylation, as observed in prostate cancer tissues [89], might be suggested in leukemogenesis. In fact, ANRIL could be involved in *cis* recruitment of Polycomb Repressive Complex 1 (PRC1) and 2 (PRC2) for the epigenetic silencing of *p14*, *p15*, and *p16*, with subsequent induction of cell cycle perturbations, differentiation block, and apoptosis arrest in blood cells, leading to leukemia.

Growth Arrest Specific 5 (GAS5) is a cell-cycle arrest and apoptosis-related lncRNA with tumor suppressor activity [90–94]. Interaction of GAS5 with the DNA binding domain of glucocorticoid receptors (GRs) leads to the suppression of glucocorticoid-mediated transcription of several antiapoptotic genes in HeLa cells [95]. GAS5 is encoded at 1q25.1 locus that has been associated with diffuse large B-cell lymphoma [92, 96] as a result of recurrent breakpoints or duplication events. GAS5 transcript levels were found significantly reduced in breast cancer [91], renal cell carcinoma [93], non-small-cell lung carcinoma [94], hepatocellular carcinoma [97], and cervical cancer tissues [98]. Interestingly, GAS5 has also been shown to be regulated by the mammalian target of rapamycin (mTOR) pathway and to mediate the effect of mTOR antagonists, such as rapamycin, on the cell cycle in T-cells [99]. Indeed, downregulation of GAS5 using RNA interference protected both leukemic and primary human T-cells from the inhibition of proliferation produced by mTOR antagonists [100]. In their recent work, Ronchetti et al. [85] found GAS5 progressively dysregulated from normal to pathological PCs through the increasingly aggressive stages of PC dyscrasia. Additionally, GAS5 was specifically upregulated in samples with 1q gain lesion. In contrast with the evidence of GAS5 overexpression in MM cells [85], a study by Isin et al. investigating the circulating levels of selected lncRNAs in plasma of patients with B-cell malignancies found that expression of GAS5 was significantly lower in the patients with MM compared to the levels in healthy subjects [101]. However, more studies are needed to fully elucidate these findings.

Another lncRNA deregulated in cancer is the taurine upregulated gene 1 (TUG1). Its expression levels were found significantly different in plasma of patients with MM compared to healthy subjects [101]. Moreover, higher levels of TUG1 correlated with disease state in both CLL and MM. TUG1 has been shown to be transcriptionally regulated by p53 in response to DNA damage [102]. Notably, it is involved in repressing important cell cycle related genes by recruiting the PRC2 complex at chromatin level. Higher TUG1 expression was also observed in bladder urothelial carcinoma samples compared to paired normal urothelium [103].

Metastasis-associated lung adenocarcinoma transcript 1 (MALAT1) is a putative oncogenic lncRNA of more than 8000 nt transcribed from chromosome 11q13.1, and overexpressed in several solid tumors including lung, colorectal, bladder and laryngeal cancers [104–107]. MALAT1 is highly conserved in mammals, a finding indicative of its potentially important function(s). MALAT1 localizes to nuclear speckles, a sub-nuclear domain suggested to coordinate RNA polymerase II transcription, pre-mRNA splicing and mRNA export [108]. Indeed, a role in the regulation of alternative splicing and cell cycle has been proposed for this lncRNA [109–111]. MALAT1 has been found to be overexpressed in MM and to represent a putative marker to predict MM progression. Specifically, Cho et al. [112] found MALAT1 expression significantly higher in MM patients at diagnosis compared to treated patients or healthy individuals. In addition, patients who experienced disease progression or relapse showed a significant increased expression of MALAT1. Of note, the expression of MALAT1 in newly diagnosed patients was not correlated with the percentage of plasma cells in the bone marrow suggesting that interaction between myeloma cells and bone marrow microenvironment may influence MALAT1 expression. Very recently, Ronchetti et al. [85] confirmed and extended this finding showing a significant overexpression of MALAT1 in myeloma PCs compared to normal PCs. Of note, the upregulation of MALAT1 appeared associated with molecular pathways involving cell cycle regulation, p53-mediated DNA damage response, and mRNA maturation processes. Differently from the evidence of MALAT1 overexpression in MM cells [85, 112], Isin et al. reported that circulating levels of MALAT1 transcripts were found to be significantly lower in patients with MM compared to healthy subjects [101]. However, these findings will require additional studies to be fully elucidated. Finally, it has been recently demonstrated that MALAT1 regulates the transcription of the nearby antisense protein-coding gene *LTBP3* (latent TGF-β-binding protein) in mesenchymal stem cells (MSCs) from MM patients [113]. LTBP3 is known to regulate the bioavailability of TGF-β, which plays an important role in the suppression of bone formation in MM bone lesions [114]. More specifically, MALAT1, expressed at high levels in the MSCs from myeloma patients, was shown to recruit the transcription factor SP1 on the *LTBP3* promoter contributing to the increase of *LTBP3* expression, most likely by stabilizing the interaction between SP1 and SP1-consensus sequences. Notably, knockdown of MALAT1 significantly decreased *LTBP3* transcription [113].

## CONCLUSIONS

An increasing number of evidences suggests that lncRNAs play central roles both in normal and malignant hematopoiesis. In this review, we summarized and discussed what is known about normally expressed and dysregulated lncRNAs during hematopoiesis with a particular focus on those lncRNAs found to modulate crucial genes in different pathways. In the next future, we expect to witness an extended identification of new functional lncRNAs expressed during all stages of normal and malignant hematopoiesis. These findings will not only contribute to better defining the role of lncRNAs in blood cell development, but might have a clinical impact. In fact, thanks to changes in their expression associated with different classes of hematological neoplasia, lncRNAs might be useful as novel biomarkers for diagnosis, prognosis and prediction of response to therapy. Furthermore, lncRNAs might also be potential therapeutic targets to be modulated, and lncRNA-based therapies could become an important health-care strategy to be considered.

## References

[1] Birney E, Stamatoyannopoulos JA, Dutta A, Guigo R, Gingeras TR, Margulies EH, Weng Z, Snyder M, Dermitzakis ET, Thurman RE, Kuehn MS, Taylor CM, Neph S (2007). Identification and analysis of functional elements in 1% of the human genome by the ENCODE pilot project. Nature.

[2] Djebali S, Davis CA, Merkel A, Dobin A, Lassmann T, Mortazavi A, Tanzer A, Lagarde J, Lin W, Schlesinger F, Xue C, Marinov GK, Khatun J (2012). Landscape of transcription in human cells. Nature.

[3] Fabian MR, Sonenberg N (2012). The mechanics of miRNA-mediated gene silencing: a look under the hood of miRISC. Nature structural & molecular biology.

[4] Kong YW, Ferland-McCollough D, Jackson TJ, Bushell M (2012). microRNAs in cancer management. The Lancet Oncology.

[5] Berindan-Neagoe I, Monroig Pdel C, Pasculli B, Calin GA (2014). MicroRNAome genome: a treasure for cancer diagnosis and therapy. CA Cancer J Clin.

[6] Garzon R, Marcucci G, Croce CM (2010). Targeting microRNAs in cancer: rationale, strategies and challenges. Nature reviews Drug discovery.

[7] Lawrie CH (2013). MicroRNAs in hematological malignancies. Blood reviews.

[8] Amodio N, Rossi M, Raimondi L, Pitari MR, Botta C, Tagliaferri P, Tassone P (2015). miR-29s: a family of epi-miRNAs with therapeutic implications in hematologic malignancies. Oncotarget.

[9] Kelley D, Rinn J (2012). Transposable elements reveal a stem cell-specific class of long noncoding RNAs. Genome biology.

[10] Johnsson P, Lipovich L, Grander D, Morris KV (2014). Evolutionary conservation of long non-coding RNAs; sequence, structure, function. Biochimica et biophysica acta.

[11] Diederichs S (2014). The four dimensions of noncoding RNA conservation. Trends in genetics.

[12] Smith MA, Gesell T, Stadler PF, Mattick JS (2013). Widespread purifying selection on RNA structure in mammals. Nucleic acids research.

[13] Hauptman N, Glavac D (2013). Long non-coding RNA in cancer. International journal of molecular sciences.

[14] Ponting CP, Oliver PL, Reik W (2009). Evolution and functions of long noncoding RNAs. Cell.

[15] Cabili MN, Trapnell C, Goff L, Koziol M, Tazon-Vega B, Regev A, Rinn JL (2011). Integrative annotation of human large intergenic noncoding RNAs reveals global properties and specific subclasses. Genes & development.

[16] Mercer TR, Mattick JS (2013). Structure and function of long noncoding RNAs in epigenetic regulation. Nature structural & molecular biology.

[17] Volders PJ, Verheggen K, Menschaert G, Vandepoele K, Martens L, Vandesompele J, Mestdagh P (2015). An update on LNCipedia: a database for annotated human lncRNA sequences. Nucleic acids research.

[18] Wang KC, Chang HY (2011). Molecular mechanisms of long noncoding RNAs. Molecular cell.

[19] Lee JT (2012). Epigenetic regulation by long noncoding RNAs. Science.

[20] Wapinski O, Chang HY (2011). Long noncoding RNAs and human disease. Trends in cell biology.

[21] Hung T, Chang HY (2010). Long noncoding RNA in genome regulation: prospects and mechanisms. RNA biology.

[22] Poliseno L, Salmena L, Zhang J, Carver B, Haveman WJ, Pandolfi PP (2010). A coding-independent function of gene and pseudogene mRNAs regulates tumour biology. Nature.

[23] Geisler S, Coller J (2013). RNA in unexpected places: long non-coding RNA functions in diverse cellular contexts. Nature reviews Molecular cell biology.

[24] Ling H, Vincent K, Pichler M, Fodde R, Berindan-Neagoe I, Slack FJ, Calin GA (2015). Junk DNA and the long non-coding RNA twist in cancer genetics. Oncogene.

[25] Yang G, Lu X, Yuan L (2014). LncRNA: a link between RNA and cancer. Biochimica et biophysica acta.

[26] Wagner LA, Christensen CJ, Dunn DM, Spangrude GJ, Georgelas A, Kelley L, Esplin MS, Weiss RB, Gleich GJ (2007). EGO, a novel, noncoding RNA gene, regulates eosinophil granule protein transcript expression. Blood.

[27] Zhang X, Lian Z, Padden C, Gerstein MB, Rozowsky J, Snyder M, Gingeras TR, Kapranov P, Weissman SM, Newburger PE (2009). A myelopoiesis-associated regulatory intergenic noncoding RNA transcript within the human HOXA cluster. Blood.

[28] Aoki K, Harashima A, Sano M, Yokoi T, Nakamura S, Kibata M, Hirose T (2010). A thymus-specific noncoding RNA, Thy-ncR1, is a cytoplasmic riboregulator of MFAP4 mRNA in immature T-cell lines. BMC molecular biology.

[29] Hu W, Yuan B, Flygare J, Lodish HF (2011). Long noncoding RNA-mediated anti-apoptotic activity in murine erythroid terminal differentiation. Genes & development.

[30] Han BW, Chen YQ (2013). Potential pathological and functional links between long noncoding RNAs and hematopoiesis. Science signaling.

[31] Yu W, Gius D, Onyango P, Muldoon-Jacobs K, Karp J, Feinberg AP, Cui H (2008). Epigenetic silencing of tumour suppressor gene p15 by its antisense RNA. Nature.

[32] Yildirim E, Kirby JE, Brown DE, Mercier FE, Sadreyev RI, Scadden DT, Lee JT (2013). Xist RNA is a potent suppressor of hematologic cancer in mice. Cell.

[33] Alvarez-Dominguez JR, Hu W, Yuan B, Shi J, Park SS, Gromatzky AA, van Oudenaarden A, Lodish HF (2014). Global discovery of erythroid long noncoding RNAs reveals novel regulators of red cell maturation. Blood.

[34] Paralkar VR, Mishra T, Luan J, Yao Y, Kossenkov AV, Anderson SM, Dunagin M, Pimkin M, Gore M, Sun D, Konuthula N, Raj A, An X (2014). Lineage and species-specific long noncoding RNAs during erythro-megakaryocytic development. Blood.

[35] Ebralidze AK, Guibal FC, Steidl U, Zhang P, Lee S, Bartholdy B, Jorda MA, Petkova V, Rosenbauer F, Huang G, Dayaram T, Klupp J, O'Brien KB (2008). PU. 1 expression is modulated by the balance of functional sense and antisense RNAs regulated by a shared cis-regulatory element. Genes & development.

[36] Dahl R, Walsh JC, Lancki D, Laslo P, Iyer SR, Singh H, Simon MC (2003). Regulation of macrophage and neutrophil cell fates by the PU. 1:C/EBPalpha ratio and granulocyte colony-stimulating factor. Nature immunology.

[37] Zhang X, Weissman SM, Newburger PE (2014). Long intergenic non-coding RNA HOTAIRM1 regulates cell cycle progression during myeloid maturation in NB4 human promyelocytic leukemia cells. RNA biology.

[38] Pang KC, Dinger ME, Mercer TR, Malquori L, Grimmond SM, Chen W, Mattick JS (1950). Genome-wide identification of long noncoding RNAs in CD8+ T cells. Journal of immunology.

[39] Sharma S, Findlay GM, Bandukwala HS, Oberdoerffer S, Baust B, Li Z, Schmidt V, Hogan PG, Sacks DB, Rao A (2011). Dephosphorylation of the nuclear factor of activated T cells (NFAT) transcription factor is regulated by an RNA-protein scaffold complex. Proceedings of the National Academy of Sciences of the United States of America.

[40] Zhang CC, Kaba M, Ge G, Xie K, Tong W, Hug C, Lodish HF (2006). Angiopoietin-like proteins stimulate ex vivo expansion of hematopoietic stem cells. Nature medicine.

[41] Vigneau S, Rohrlich PS, Brahic M, Bureau JF (2003). Tmevpg1, a candidate gene for the control of Theiler's virus persistence, could be implicated in the regulation of gamma interferon. Journal of virology.

[42] Collier SP, Collins PL, Williams CL, Boothby MR, Aune TM (1950). Cutting edge: influence of Tmevpg1, a long intergenic noncoding RNA, on the expression of Ifng by Th1 cells. Journal of immunology.

[43] Gomez JA, Wapinski OL, Yang YW, Bureau JF, Gopinath S, Monack DM, Chang HY, Brahic M, Kirkegaard K (2013). The NeST long ncRNA controls microbial susceptibility and epigenetic activation of the interferon-gamma locus. Cell.

[44] Ranzani V, Rossetti G (2015). The long intergenic noncoding RNA landscape of human lymphocytes highlights the regulation of T cell differentiation by linc-MAF-4.

[45] Eis PS, Tam W, Sun L, Chadburn A, Li Z, Gomez MF, Lund E, Dahlberg JE (2005). Accumulation of miR-155 and BIC RNA in human B cell lymphomas. Proceedings of the National Academy of Sciences of the United States of America.

[46] Tam W (2001). Identification and characterization of human BIC, a gene on chromosome 21 that encodes a noncoding RNA. Gene.

[47] Elton TS, Selemon H, Elton SM, Parinandi NL (2013). Regulation of the MIR155 host gene in physiological and pathological processes. Gene.

[48] Landgraf P, Rusu M, Sheridan R, Sewer A, Iovino N, Aravin A, Pfeffer S, Rice A, Kamphorst AO, Landthaler M, Lin C, Socci ND, Hermida L (2007). A mammalian microRNA expression atlas based on small RNA library sequencing. Cell.

[49] Calin GA, Liu CG, Ferracin M, Hyslop T, Spizzo R, Sevignani C, Fabbri M, Cimmino A, Lee EJ, Wojcik SE, Shimizu M, Tili E, Rossi S (2007). Ultraconserved regions encoding ncRNAs are altered in human leukemias and carcinomas. Cancer cell.

[50] Petri A, Dybkaer K, Bogsted M, Thrue CA, Hagedorn PH, Schmitz A, Bodker JS, Johnsen HE, Kauppinen S (2015). Long Noncoding RNA Expression during Human B-Cell Development. PloS one.

[51] Chaligne R, Heard E (2014). X-chromosome inactivation in development and cancer. FEBS letters.

[52] Gabory A, Jammes H, Dandolo L (2010). The H19 locus: role of an imprinted non-coding RNA in growth and development. BioEssays.

[53] Bock O, Schlue J, Kreipe H (2003). Reduced expression of H19 in bone marrow cells from chronic myeloproliferative disorders. Leukemia.

[54] Tessema M, Langer F, Bock O, Seltsam A, Metzig K, Hasemeier B, Kreipe H, Lehmann U (2005). Down-regulation of the IGF-2/H19 locus during normal and malignant hematopoiesis is independent of the imprinting pattern. International journal of oncology.

[55] Guo G, Kang Q, Chen Q, Chen Z, Wang J, Tan L, Chen JL (2014). High expression of long non-coding RNA H19 is required for efficient tumorigenesis induced by Bcr-Abl oncogene. FEBS letters.

[56] Takeuchi S, Hofmann WK, Tsukasaki K, Takeuchi N, Ikezoe T, Matsushita M, Uehara Y, Phillip Koeffler H (2007). Loss of H19 imprinting in adult T-cell leukaemia/lymphoma. British journal of haematology.

[57] Cai X, Cullen BR (2007). The imprinted H19 noncoding RNA is a primary microRNA precursor. RNA.

[58] Tsang WP, Ng EK, Ng SS, Jin H, Yu J, Sung JJ, Kwok TT (2010). Oncofetal H19-derived miR-675 regulates tumor suppressor RB in human colorectal cancer. Carcinogenesis.

[59] Guo G, Kang Q, Zhu X, Chen Q, Wang X, Chen Y, Ouyang J, Zhang L, Tan H, Chen R, Huang S, Chen JL (2015). A long noncoding RNA critically regulates Bcr-Abl-mediated cellular transformation by acting as a competitive endogenous RNA. Oncogene.

[60] Sun J, Li W, Sun Y, Yu D, Wen X, Wang H, Cui J, Wang G, Hoffman AR, Hu JF (2014). A novel antisense long noncoding RNA within the IGF1R gene locus is imprinted in hematopoietic malignancies. Nucleic acids research.

[61] Chapuis N, Tamburini J, Cornillet-Lefebvre P, Gillot L, Bardet V, Willems L, Park S, Green AS, Ifrah N, Dreyfus F, Mayeux P, Lacombe C, Bouscary D (2010). Autocrine IGF-1/IGF-1R signaling is responsible for constitutive PI3K/Akt activation in acute myeloid leukemia: therapeutic value of neutralizing anti-IGF-1R antibody. Haematologica.

[62] Zhou Y, Zhong Y, Wang Y, Zhang X, Batista DL, Gejman R, Ansell PJ, Zhao J, Weng C, Klibanski A (2007). Activation of p53 by MEG3 non-coding RNA. The Journal of biological chemistry.

[63] Benetatos L, Hatzimichael E, Dasoula A, Dranitsaris G, Tsiara S, Syrrou M, Georgiou I, Bourantas KL (2010). CpG methylation analysis of the MEG3 and SNRPN imprinted genes in acute myeloid leukemia and myelodysplastic syndromes. Leukemia research.

[64] Khoury H, Suarez-Saiz F, Wu S, Minden MD (2010). An upstream insulator regulates DLK1 imprinting in AML. Blood.

[65] Benetatos L, Dasoula A, Hatzimichael E, Georgiou I, Syrrou M, Bourantas KL (2008). Promoter hypermethylation of the MEG3 (DLK1/MEG3) imprinted gene in multiple myeloma. Clinical lymphoma & myeloma.

[66] Zhang X, Gejman R, Mahta A, Zhong Y, Rice KA, Zhou Y, Cheunsuchon P, Louis DN, Klibanski A (2010). Maternally expressed gene 3, an imprinted noncoding RNA gene, is associated with meningioma pathogenesis and progression. Cancer research.

[67] Benetatos L, Vartholomatos G, Hatzimichael E (2011). MEG3 imprinted gene contribution in tumorigenesis. International journal of cancer.

[68] Garzon R, Volinia S, Papaioannou D, Nicolet D, Kohlschmidt J, Yan PS, Mrozek K, Bucci D, Carroll AJ, Baer MR, Wetzler M, Carter TH, Powell BL (2014). Expression and prognostic impact of lncRNAs in acute myeloid leukemia. Proceedings of the National Academy of Sciences of the United States of America.

[69] Diaz-Beya M, Brunet S, Nomdedeu J, Pratcorona M, Cordeiro A, Gallardo D, Escoda L, Tormo M, Heras I, Ribera JM, Duarte R, de Llano MP, Bargay J (2015). The lincRNA HOTAIRM1, located in the HOXA genomic region, is expressed in acute myeloid leukemia, impacts prognosis in patients in the intermediate-risk cytogenetic category, and is associated with a distinctive microRNA signature. Oncotarget.

[70] Tanigaki K, Honjo T (2007). Regulation of lymphocyte development by Notch signaling. Nature immunology.

[71] Tzoneva G, Ferrando AA (2012). Recent advances on NOTCH signaling in T-ALL. Current topics in microbiology and immunology.

[72] Trimarchi T, Bilal E, Ntziachristos P, Fabbri G, Dalla-Favera R, Tsirigos A, Aifantis I (2014). Genome-wide mapping and characterization of Notch-regulated long noncoding RNAs in acute leukemia. Cell.

[73] Medyouf H, Gusscott S, Wang H, Tseng JC, Wai C, Nemirovsky O, Trumpp A, Pflumio F, Carboni J, Gottardis M, Pollak M, Kung AL, Aster JC (2011). High-level IGF1R expression is required for leukemia-initiating cell activity in T-ALL and is supported by Notch signaling. The Journal of experimental medicine.

[74] Gaidano G, Foa R, Dalla-Favera R (2012). Molecular pathogenesis of chronic lymphocytic leukemia. The Journal of clinical investigation.

[75] Dohner H, Stilgenbauer S, Benner A, Leupolt E, Krober A, Bullinger L, Dohner K, Bentz M, Lichter P (2000). Genomic aberrations and survival in chronic lymphocytic leukemia. The New England journal of medicine.

[76] Cimmino A, Calin GA, Fabbri M, Iorio MV, Ferracin M, Shimizu M, Wojcik SE, Aqeilan RI, Zupo S, Dono M, Rassenti L, Alder H, Volinia S (2005). miR-15 and miR-16 induce apoptosis by targeting BCL2. Proceedings of the National Academy of Sciences of the United States of America.

[77] Calin GA, Ferracin M, Cimmino A, Di Leva G, Shimizu M, Wojcik SE, Iorio MV, Visone R, Sever NI, Fabbri M, Iuliano R, Palumbo T, Pichiorri F (2005). A MicroRNA signature associated with prognosis and progression in chronic lymphocytic leukemia. The New England journal of medicine.

[78] Klein U, Lia M, Crespo M, Siegel R, Shen Q, Mo T, Ambesi-Impiombato A, Califano A, Migliazza A, Bhagat G, Dalla-Favera R (2010). The DLEU2/miR-15a/16-1 cluster controls B cell proliferation and its deletion leads to chronic lymphocytic leukemia. Cancer cell.

[79] Lerner M, Harada M, Loven J, Castro J, Davis Z, Oscier D, Henriksson M, Sangfelt O, Grander D, Corcoran MM (2009). DLEU2, frequently deleted in malignancy, functions as a critical host gene of the cell cycle inhibitory microRNAs miR-15a and miR-16-1. Experimental cell research.

[80] Garding A, Bhattacharya N, Claus R, Ruppel M, Tschuch C, Filarsky K, Idler I, Zucknick M, Caudron-Herger M, Oakes C, Fleig V, Keklikoglou I, Allegra D (2013). Epigenetic upregulation of lncRNAs at 13q14. 3 in leukemia is linked to the In Cis downregulation of a gene cluster that targets NF-kB. PLoS genetics.

[81] Kohlhammer H, Schwaenen C, Wessendorf S, Holzmann K, Kestler HA, Kienle D, Barth TF, Moller P, Ott G, Kalla J, Radlwimmer B, Pscherer A, Stilgenbauer S (2004). Genomic DNA-chip hybridization in t(11;14)-positive mantle cell lymphomas shows a high frequency of aberrations and allows a refined characterization of consensus regions. Blood.

[82] Bigoni R, Cuneo A, Milani R, Roberti MG, Bardi A, Rigolin GM, Cavazzini F, Agostini P, Castoldi G (2001). Secondary chromosome changes in mantle cell lymphoma: cytogenetic and fluorescence in situ hybridization studies. Leukemia & lymphoma.

[83] Harrison CJ, Mazzullo H, Cheung KL, Gerrard G, Jalali GR, Mehta A, Osier DG, Orchard KH (2003). Cytogenetics of multiple myeloma: interpretation of fluorescence in situ hybridization results. British journal of haematology.

[84] Elnenaei MO, Hamoudi RA, Swansbury J, Gruszka-Westwood AM, Brito-Babapulle V, Matutes E, Catovsky D (2003). Delineation of the minimal region of loss at 13q14 in multiple myeloma. Genes, chromosomes & cancer.

[85] Ronchetti D, Agnelli L, Taiana E, Galletti S, Manzoni M, Todoerti K, Musto P, Strozzi F, Neri A (2016). Distinct lncRNA transcriptional fingerprints characterize progressive stages of multiple myeloma. Oncotarget.

[86] Popov N, Gil J (2010). Epigenetic regulation of the INK4b-ARF-INK4a locus: in sickness and in health. Epigenetics.

[87] Drexler HG (1998). Review of alterations of the cyclin-dependent kinase inhibitor INK4 family genes p15, p16, p18 and p19 in human leukemia-lymphoma cells. Leukemia.

[88] Iacobucci I, Sazzini M, Garagnani P, Ferrari A, Boattini A, Lonetti A, Papayannidis C, Mantovani V, Marasco E, Ottaviani E, Soverini S, Girelli D, Luiselli D (2011). A polymorphism in the chromosome 9p21 ANRIL locus is associated to Philadelphia positive acute lymphoblastic leukemia. Leukemia research.

[89] Yap KL, Li S, Munoz-Cabello AM, Raguz S, Zeng L, Mujtaba S, Gil J, Walsh MJ, Zhou MM (2010). Molecular interplay of the noncoding RNA ANRIL and methylated histone H3 lysine 27 by polycomb CBX7 in transcriptional silencing of INK4a. Molecular cell.

[90] Coccia EM, Cicala C, Charlesworth A, Ciccarelli C, Rossi GB, Philipson L, Sorrentino V (1992). Regulation and expression of a growth arrest-specific gene (gas5) during growth, differentiation, and development. Molecular and cellular biology.

[91] Mourtada-Maarabouni M, Pickard MR, Hedge VL, Farzaneh F, Williams GT (2009). GAS5, a non-protein-coding RNA, controls apoptosis and is downregulated in breast cancer. Oncogene.

[92] Nakamura Y, Takahashi N, Kakegawa E, Yoshida K, Ito Y, Kayano H, Niitsu N, Jinnai I, Bessho M (2008). The GAS5 (growth arrest-specific transcript 5) gene fuses to BCL6 as a result of t(1;3)(q25;q27) in a patient with B-cell lymphoma. Cancer genetics and cytogenetics.

[93] Qiao HP, Gao WS, Huo JX, Yang ZS (2013). Long non-coding RNA GAS5 functions as a tumor suppressor in renal cell carcinoma. Asian Pacific journal of cancer prevention.

[94] Shi X, Sun M, Liu H, Yao Y, Kong R, Chen F, Song Y (2015). A critical role for the long non-coding RNA GAS5 in proliferation and apoptosis in non-small-cell lung cancer. Molecular carcinogenesis.

[95] Kino T, Hurt DE, Ichijo T, Nader N, Chrousos GP (2010). Noncoding RNA gas5 is a growth arrest- and starvation-associated repressor of the glucocorticoid receptor. Science signaling.

[96] Dave BJ, Nelson M, Pickering DL, Chan WC, Greiner TC, Weisenburger DD, Armitage JO, Sanger WG (2002). Cytogenetic characterization of diffuse large cell lymphoma using multi-color fluorescence in situ hybridization. Cancer genetics and cytogenetics.

[97] Tu ZQ, Li RJ, Mei JZ, Li XH (2014). Down-regulation of long non-coding RNA GAS5 is associated with the prognosis of hepatocellular carcinoma. International journal of clinical and experimental pathology.

[98] Cao S, Liu W, Li F, Zhao W, Qin C (2014). Decreased expression of lncRNA GAS5 predicts a poor prognosis in cervical cancer. International journal of clinical and experimental pathology.

[99] Williams GT, Mourtada-Maarabouni M, Farzaneh F (2011). A critical role for non-coding RNA GAS5 in growth arrest and rapamycin inhibition in human T-lymphocytes. Biochemical Society transactions.

[100] Mourtada-Maarabouni M, Hasan AM, Farzaneh F, Williams GT (2010). Inhibition of human T-cell proliferation by mammalian target of rapamycin (mTOR) antagonists requires noncoding RNA growth-arrest-specific transcript 5 (GAS5). Molecular pharmacology.

[101] Isin M, Ozgur E, Cetin G, Erten N, Aktan M, Gezer U, Dalay N (2014). Investigation of circulating lncRNAs in B-cell neoplasms. Clinica chimica acta.

[102] Khalil AM, Guttman M, Huarte M, Garber M, Raj A, Rivea Morales D, Thomas K, Presser A, Bernstein BE, van Oudenaarden A, Regev A, Lander ES, Rinn JL (2009). Many human large intergenic noncoding RNAs associate with chromatin-modifying complexes and affect gene expression. Proceedings of the National Academy of Sciences of the United States of America.

[103] Han Y, Liu Y, Gui Y, Cai Z (2013). Long intergenic non-coding RNA TUG1 is overexpressed in urothelial carcinoma of the bladder. Journal of surgical oncology.

[104] Schmidt LH, Spieker T, Koschmieder S, Schaffers S, Humberg J, Jungen D, Bulk E, Hascher A, Wittmer D, Marra A, Hillejan L, Wiebe K, Berdel WE (2011). The long noncoding MALAT-1 RNA indicates a poor prognosis in non-small cell lung cancer and induces migration and tumor growth. Journal of thoracic oncology.

[105] Xu C, Yang M, Tian J, Wang X, Li Z (2011). MALAT-1: a long non-coding RNA and its important 3′ end functional motif in colorectal cancer metastasis. International journal of oncology.

[106] Ying L, Chen Q, Wang Y, Zhou Z, Huang Y, Qiu F (2012). Upregulated MALAT-1 contributes to bladder cancer cell migration by inducing epithelial-to-mesenchymal transition. Molecular bioSystems.

[107] Gutschner T, Hammerle M, Eissmann M, Hsu J, Kim Y, Hung G, Revenko A, Arun G, Stentrup M, Gross M, Zornig M, MacLeod AR, Spector DL (2013). The noncoding RNA MALAT1 is a critical regulator of the metastasis phenotype of lung cancer cells. Cancer research.

[108] Bernard D, Prasanth KV, Tripathi V, Colasse S, Nakamura T, Xuan Z, Zhang MQ, Sedel F, Jourdren L, Coulpier F, Triller A, Spector DL, Bessis A (2010). A long nuclear-retained non-coding RNA regulates synaptogenesis by modulating gene expression. The EMBO journal.

[109] Tripathi V, Ellis JD, Shen Z, Song DY, Pan Q, Watt AT, Freier SM, Bennett CF, Sharma A, Bubulya PA, Blencowe BJ, Prasanth SG, Prasanth KV (2010). The nuclear-retained noncoding RNA MALAT1 regulates alternative splicing by modulating SR splicing factor phosphorylation. Molecular cell.

[110] Yang F, Yi F, Han X, Du Q, Liang Z (2013). MALAT-1 interacts with hnRNP C in cell cycle regulation. FEBS letters.

[111] Tripathi V, Shen Z, Chakraborty A, Giri S, Freier SM, Wu X, Zhang Y, Gorospe M, Prasanth SG, Lal A, Prasanth KV (2013). Long noncoding RNA MALAT1 controls cell cycle progression by regulating the expression of oncogenic transcription factor B-MYB. PLoS genetics.

[112] Cho SF, Chang YC, Chang CS, Lin SF, Liu YC, Hsiao HH, Chang JG, Liu TC (2014). MALAT1 long non-coding RNA is overexpressed in multiple myeloma and may serve as a marker to predict disease progression. BMC cancer.

[113] Li B, Chen P, Qu J, Shi L, Zhuang W, Fu J, Li J, Zhang X, Sun Y, Zhuang W (2014). Activation of LTBP3 gene by a long noncoding RNA (lncRNA) MALAT1 transcript in mesenchymal stem cells from multiple myeloma. The Journal of biological chemistry.

[114] Matsumoto T, Abe M (2011). TGF-beta-related mechanisms of bone destruction in multiple myeloma. Bone.

[115] Dal Bo M, Rossi FM, Rossi D, Deambrogi C, Bertoni F, Del Giudice I, Palumbo G, Nanni M, Rinaldi A, Kwee I, Tissino E, Corradini G, Gozzetti A (2011). 13q14 deletion size and number of deleted cells both influence prognosis in chronic lymphocytic leukemia. Genes, chromosomes & cancer.

[116] Hutchinson JN, Ensminger AW, Clemson CM, Lynch CR, Lawrence JB, Chess A (2007). A screen for nuclear transcripts identifies two linked noncoding RNAs associated with SC35 splicing domains. BMC genomics.

[117] Miyagawa R, Tano K, Mizuno R, Nakamura Y, Ijiri K, Rakwal R, Shibato J, Masuo Y, Mayeda A, Hirose T, Akimitsu N (2012). Identification of cis- and trans-acting factors involved in the localization of MALAT-1 noncoding RNA to nuclear speckles. RNA.

